# Possible Antidepressant Effects of Memantine—Systematic Review with a Case Study

**DOI:** 10.3390/ph14050481

**Published:** 2021-05-18

**Authors:** Marek Krzystanek, Stanisław Surma, Artur Pałasz, Monika Romańczyk, Krzysztof Krysta

**Affiliations:** 1 Department of Psychiatry and Psychotherapy, Clinic of Psychiatric Rehabilitation, Faculty of Medical Sciences in Katowice, Medical University of Silesia, Ziołowa 45/47, 40-635 Katowice, Poland; stasiu.surma@onet.eu (S.S.); romanczykmonika@wp.pl (M.R.); Ochojec@gmail.com (K.K.); 2 Department of Histology, Faculty of Medical Sciences in Katowice, Medical University of Silesia, Medyków 18, 40-752 Katowice, Poland; artiassone@gmail.com

**Keywords:** memantine, bipolar depression, bipolar disorder, augmentation, mood stabilizers, amantadine derivative

## Abstract

The treatment of bipolar depression is hampered by the inadequate efficacy of antidepressants, moderate effect of mood stabilizers, and the side effects of some second-generation antipsychotics. There is limited evidence to date regarding the antidepressant effects of memantine in bipolar depression. The aim of the article was to provide a short review of preclinical and clinical studies on the antidepressant effect of memantine, and to present the case of a bipolar depression patient successfully treated with memantine. The described patient with bipolar disorder was unsuccessfully treated with two mood stabilizers. The addition of memantine at a dose of 20 mg/d to the treatment with lamotrigine and valproic acid resulted in a reduction in the severity of depression measured on the HDRS-17 scale by 35%, and by 47.1% after 7 weeks. The discussion presents experimental evidence for the antidepressant effect of memantine, as well as data from clinical trials in recurrent and bipolar depression. The presented case is the second report in the medical literature showing the antidepressant effect of memantine as an add-on treatment for bipolar depression. The described case and literature analysis indicate that memantine may be an effective and safe method of augmentation of mood stabilizing therapy in bipolar depression.

## 1. Introduction

Treating depression in bipolar disorder (BD) is a difficult clinical task. One of the main problems in treating depression in bipolar disorder is, on the one hand, the ineffectiveness of antidepressants and, on the other hand, the moderate antidepressant efficacy of mood-stabilizing drugs [1]. Additionally, the use of antidepressants in bipolar depression is generally contraindicated, because they may increase cycle acceleration, causing a rapid cycling course, and contribute to the formation of mixed episodes [1,2]. Attempts to treat bipolar depression with second-generation antipsychotic drugs such as quetiapine and olanzapine may in turn be associated with the risk of deterioration of the functioning of patients, caused by the occurrence of metabolic, motoric, and cognitive side effects; however, the effects of using in aripiprazole and lurasidone are promising in this indication [2]. For this reason, it is imperative to search for new drugs to treat bipolar depression.

Memantine, as well as the structurally analogous adamantane derivative of amantadine [3], is an uncompetitive, low-affinity, and selective open-channel blocker of NMDA receptor (NMDAR). The mechanism of action of memantine is very complex, which may indicate its potential in regulating processes in the central nervous system and, consequently, in the treatment of mental disorders.

Because the relatively fast off-rate accumulation of memantine in the channel pore does not occur, the synaptic transmission is not substantially affected [4]. Unlike ketamine, another and well-known NMDAR channel blocker, memantine does not exhibit serious side effects and its inhibitory effect seems to be stereospecific. Memantine binding to the channel pore is considered voltage-independent, and its molecule forms hydrogen bonds with the N + 1 asparagine residue (N613 of GluN2B.) [5]. Because alterations in the NMDAR activity can modulate neuronal excitability, even limited differences in the channel blockers’ modes of action can distinctly change their clinical properties [6]. The blockade of presynaptic NMDARs in the dopaminergic neurons inhibits dopamine transporter (DAT1) activity and subsequently indirectly increases the dopamine level within the synaptic cleft. Moreover, memantine enhances hippocampal long-term potentiation (LTP) in mice by an increase in CaMKII activity and inhibits ATP-sensitive inward-rectifier K_ir_ 6.1 and K_ir_ 6.2 potassium channels in the cultured neurons [7].

**Figure 1:**
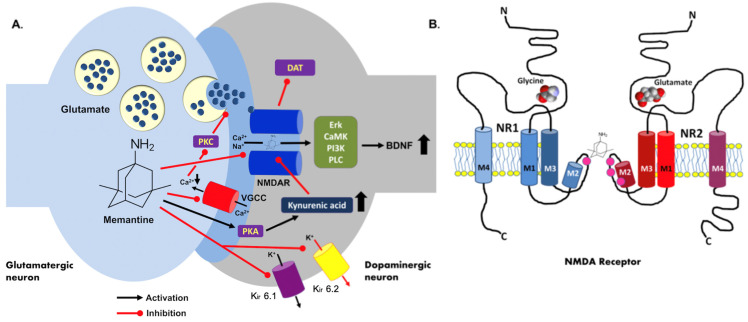
Molecular mechanism of memantine action. (**A**) The memantine molecule acts as a blocker of the NMDAR ionic channel and it is able to regulate the synaptic dopamine concentration via the inhibition of the presynaptic dopamine transporter (DAT1) activity. Moreover, the blockade of the NMDAR function causes an upregulation of BDNF expression via the Erk, CamK, PI3K, and PLC signaling pathways. Memantine can inhibit inwardly rectifying potassium channels K_ir_ 6.1 and K_ir_ 6.2, and may also decrease voltage-gated calcium channels (VGCC)-dependent glutamate exocytosis. A synthesis of kynurenic acid is stimulated by memantine through the activation of protein kinase A (PKA). (**B**) Schematic NMDAR structure in the context of memantine action. Two receptor subunits (NR1 and NR2) are presented (transmembrane domains marked in blue and red, respectively) with their glycine and glutamate binding sites (ligand molecules included). Asparagine residues involved in channel blockade by Mg^2+^ and several inhibitors including memantine are shown. Based on Sing et al. (2018), modified. NMDAR—NMDA receptor; PKA—protein kinase A; PKC—protein kinase C; PLC—phospholipase C; Er—extracellular signal-regulated kinases; BDNF—brain derived neurotrophic factor; PI3K—phosphoinositide 3-kinase; CamK—calcium/calmodulin-dependent protein kinase. Up and down thick arrows indicate the direction of changes.

A stimulatory effect of memantine on histamine neurons was also reported with up-regulations of brain-derived neurotrophic factor (BDNF) and NMDAR NR1 subunit mRNAs in the hippocampus. The gene expression of α7-nicotinic receptors remained unaffected in any brain structure [8]. It is important to note that memantine may also reduce glutamate release, probably via the inhibition of presynaptic voltage-gated calcium channels (VGCC) and the subsequent blockade of protein kinase C (PKC)-dependent signaling [9]. The drug potently supports the synthesis of kynurenic acid through the activation of protein kinase A (PKA) shown in cortical rat brain slices. The same effect was also described in mixed glial cultures. Kynurenic acid, a neuronal tryptophan derivative, is a blocker of the NMDAR glycine site and an antagonist of nicotinic alpha7 receptors [10]. Noteworthy, memantine highly increases neural progenitor proliferation and stimulates adult neurogenesis in the mouse hippocampus, and it may reduce the activation of microglia and amyloidogenesis in 13-month-old APP/PS1 mice [11]. A summary of the memantine mechanisms of action is shown in Figure 1.

The meta-analysis by Kishi et al. published in 2017 and including six randomized clinical trials, assessed the efficacy of memantine in the treatment of depression [12]. This meta-analysis covered 451 patients with major depressive disorders—four trials (*n* = 189), three of which studied memantine augmentation for antidepressants or bipolar disorder, and two trials (*n* = 262), both on memantine augmentation for mood stabilizers. The mean study duration was 8.33 weeks, and the mean age of patients was 39.9 years. The meta-analysis showed that memantine was not superior to placebo with regard to the response rate (RR = 0.92, 95% CI = 0.70–1.20, I2 = 72%), remission rate (major depressive disorder: RR 0.94; (95% CI 0.62–1.42, I2 = 80%); bipolar disorder: RR 0.83; (95% CI 0.57–1.19, I2 = 36%)), improvement of depressive symptoms scale score, and all-cause discontinuation (RR = 0.84, 95% CI = 0.60–1.18, I2 = 0%). In conclusion, the authors stated that memantine does not improve the treatment efficacy for depressive symptoms in major depressive disorders and bipolar disorder patients [12].

Despite this, there are studies in the literature that show the possible antidepressant effect of memantine in bipolar depression [13,14,15,16]. We recently published a case series study showing the effectiveness of treating bipolar depression with amantadine [17]. Now, we would like to present a case in which amantadine was not effective and the patient was successfully treated with memantine—a drug similar both structurally and in the mechanism of action to amantadine. Moreover, to justify the use of memantine in BD and to help interpret the results of memantine treatment in the case report, we also decided to briefly review the preclinical and clinical evidence for the antidepressant effects of memantine, paying particular attention to the treatment of the depressive phase of BD.

## 2. Results

### 2.1. Preclinical Studies of Antidepressant Effect of Memantine

Summary of the preclinical evidence of the antidepressant effect of memantine was presented in Table 1.

### 2.2. Clinical Studies on Antidepressant Effect of Memantine

Summary of clinical evidence of antidepressant effect of memantine was presented in Table 2.

### 2.3. Individual Case Report

The patient used was 43 years old, and had been suffering from BD since the age of 34. The onset of the disease was not associated with distress, somatic disease, or taking psychoactive substances. When the symptoms of elevated mood and increased drive began, the patient began to abuse amphetamines, but it was already during mania. The patient stopped coming home from work, had new plans, risky money invested, and he also established extra-marital sexual relationships. Mania was diagnosed according to ICD-10. As a result of treatment with quetiapine (600 mg/d), the symptoms of mania were resolved, the patient took it at a dose of 300 mg for 3 years, and then, in consultation with the doctor, he gradually stopped taking the drug. In the spring of 2017, there was a short 3-day period of hypomania—the patient prolonged his sleep with estazolam in a dose of 2 mg at night for 1 week and the symptoms subsided.

In October 2018, the patient developed hypomania (diagnosis according to ICD-10). The patient was recommended to extend his sleep to 11 h, and he was given estazolam 2 mg at night for 2 weeks. The symptoms resolved, but after a month the patient presented with symptoms of moderate depression (HDRS 18 points). He was given quetiapine again at a target dose of 300 mg at night. After 2 months, his symptoms remained at a similar level (HDRS 18 points). Because of lithium intolerance (nausea and vomiting, a feeling of bitterness in the mouth), he then additionally received lamotrigine at a target dose of 150 mg/day. After another two months, there was no improvement (HDRS 19). It was decided to change the treatment—quetiapine was discontinued and replaced with aripiprazole at a dose of 7.5 mg. After another 6 weeks, no improvement was achieved (HDRS 18 points) and the patient continued treatment with lamotrigine 150 mg and aripiprazole 7.5 mg for another 6 weeks. As a result of the lack of improvement (HDRS 19), aripiprazole was discontinued and valproic acid was added to the treatment, titrated-up to 1500 mg/day. At the next visit after 3 months, the patient still had a depressive episode of a similar intensity as before (HDRS 18 points). The patient then consented to treatment with amantadine. Initially, he took 100 mg in the morning and 200 mg in the morning after a week. After about 10 days on a dose of 200 mg, the patient felt a slight improvement (HDRS 15) and the treatment was continued for another month. After one month, the HDRS was 17 points (Table 1).

**Figure 2:**
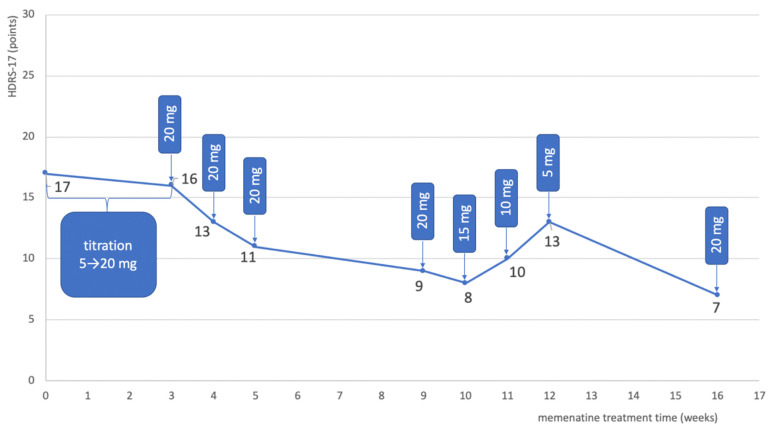
Change in the severity of depression in the bipolar depression patient, measured with the HDRS-17 scale during treatment, depending on the duration of treatment and the dose of memantine.

Amantadine was discontinued and treatment with memantine was proposed, at a target dose of 20 mg. The patient was informed about the mechanism of action of memantine, its potential side effects, and the off-label use of the drug before starting it. The patient consented to add-on treatment with memantine. The dose was increased gradually by 5 mg every 3 days. After 2 weeks on a dose of 20 mg, the patient reported feeling a little better, but the psychiatric evaluation of the HDRS was 16 points. After another week, the total score on HDRS-17 decreased to 13 points, and after another week to 11 points. When he came for an appointment after another month of treatment, the HDRS was 9 points. The patient reported that he had not felt so well in a long time. A weekly reduction of memantine was started by 5 mg, but the patient started to feel worse on the 5 mg dose (HDRS 13) and the dose was then increased to 10 mg. At the next visit after one month, the HDRS score was 7 points (Table 3 and Figure 2). Treatment did not change since then. From the beginning of the treatment, the patient tolerated the treatment with memantine well and no side effects were observed. The patient is still in stable remission.

**Figure 3:**
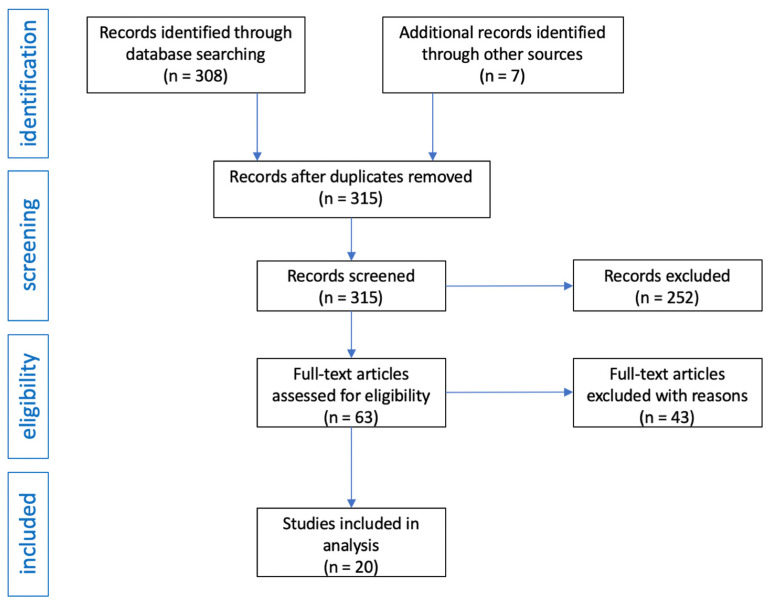
Preferred reporting items for the systematic reviews flow diagram.

Adding memantine to the patient’s for treatment with two mood stabilizers resulted in an improvement and subsequent relief of depressive symptoms. Table 3 and Figure 3 show the course of treatment and changes in the severity of depression measured with HDRS-17 during the treatment of a BD patient with an episode of bipolar depression.

## 3. Discussion

All nine experimental studies conducted so far on animal models of depression have shown the antidepressant effect of memantine [18,19,20,21,22,23,24,25,26]. It is therefore surprising that most clinical trials carried out later failed to confirm the antidepressant effect of memantine in monotherapy and as an add-on treatment [13,27,30,31]. Despite the negative results of the meta-analysis conducted in 2017 by Kish et al., there are publications pointing to the possible antidepressant effect of memantine in augmenting the treatment of recurrent depression [28,29,32]; however, they may be related to certain subtypes of depression, e.g., in the course of alcohol dependence [29] or in the improvement of symptoms of cognitive impairment in depression [33]. Importantly, there is a lack of new clinical trials on the efficacy of memantine in depression, and most of the studies presented are from 5 years ago.

In bipolar depression, memantine has been shown to be effective in the dose titration-up stage [13]. A study by Stevens et al. showed that memantine accelerates the action of lamotrigine [16]. One study also showed that the effectiveness of add-on treatment with memantine is effective in patients with Val66Met polymorphism in the BDNF gene [14]. In 2013, Strzelecki et al. described the case of a patient suffering from bipolar depression, in which memantine proved to be an effective adjunct treatment [15]. This is the only published case of a bipolar depression patient who has been shown to be successfully treated with memantine. The case report we publish supports the clinical efficacy of memantine described by Strzelecki et al.

Despite the overwhelming evidence that memantine does not have an antidepressant effect, our literature review and the reported case still suggest that memantine may nevertheless have some antidepressant effects, particularly in the treatment of bipolar depression in combination with mood stabilizing treatment. Combined with the inconsistency of preclinical and clinical data, it justifies further clinical studies on the possibility of using memantine in the treatment of depression.

In the described by Strzelecki et al., the case study for memantine showed an antidepressant effect when combined with lithium (1 g/day), olanzapine (7.5 mg/day), and mianserin (60 mg/day). Before adding memantine, this patient took lithium, olanzapine, and mianserin for several weeks without improving his depressive symptoms. It was similar in the case described by us—the patient was unsuccessfully treated for depression with lamotrigine, valproic acid, and amantadine when he started taking memantine. Perhaps memantine in bipolar depression has an antidepressant effect not in monotherapy, but as a method of augmentation of treatment with mood stabilizers. In the studies of bipolar depression that showed a certain antidepressant effect of memantine, it was added to a mood stabilizer [13,14,15,16]. This indicates the direction of further clinical trials of memantine as an add-on treatment for mood stabilizing treatment.

In the case described by Strzelecki et al., improvement occurred rapidly at the start of treatment with memantine (at 5 mg, the HDRS score fell by 21% after one week). This is a similar effect seen in the study by Anand et al., where improvement was (only) seen during the titration-up of the memantine dose [13]. However, it cannot be ruled out that the rapid improvement observed at the start of treatment may also have been a placebo effect. In the patient described by us, the titration-up was faster than in the Strzelecki study, and the improvement after 2 weeks of using amantadine in a dose of 20 mg was 35% in HDRS compared with 15.8% in the study by Strzelecki et al. The faster dose escalation was well tolerated and perhaps led to a better therapeutic effect after 2 weeks with the target dose of memantine. Interestingly, in the case described by Strzelecki et al., the improvement after 8 weeks of treatment (reduction in HDRS-17 by 47.4%) was very similar to the improvement obtained after 7 weeks of using the 20 mg dose (reduction in the HDRS score by 47.1%) in the patient described by us. Data from both case studies may indicate efficacy of the 20 mg dose of memantine in the treatment of bipolar depression. In both cases, treatment with memantine was well tolerated.

In our case, the attempt to stop taking memantine caused a deterioration of mood. In the case described by Strzelecki et al., the patient was still in remission of depressive symptoms after the discontinuation of memantine and 6 weeks later, but the authors did not follow-up the patient longer, so it is not known if the improvement was permanent. Perhaps each case must be treated individually and each time the doctor must decide whether to stop or maintain the treatment with memantine. Moreover, as there is some evidence of the anti-manic and mood-stimulating effects of memantine, it may be possible that continued treatment with memantine for an extended period of time may have a preventive effect on relapse in BD [16,34,35,36]. In order to confirm this hypothesis, a clinical trial to assess the effect of the long-term use of memantine on the frequency of relapse of BD episodes is required.

## 4. Methods

The authors decided to review the literature, considering clinical trials, case series, and case reports. PRISMA guidelines were used when preparing this systematic review [37]. Full-text publications available in English were included in the analysis. In each study, at least the baseline and endpoints of the treatment efficacy had to be characterized. The review was conducted independently by two investigators, then their search results were combined and duplicate records were removed.

The following medical databases were searched in the study: PubMed, Scopus, Web of Science, and Google Scholar (effective date 26 March 2021). The search was performed according to the PICO framework (P—patient, problem, or population; I—intervention; C—comparison, control, or comparator; O—outcomes). During our search, we used the following terms: “bipolar depression” (Title/Abstract), “memantine” (Title/Abstract), “mood stabilizers” (Title/Abstract), “improvement of depressive symptoms” (Title/Abstract), “memantine and depression” (Title/Abstract), “memantine and depression treatment” (Title/Abstract), “monotherapy” (Title/Abstract), and “augmentation” (Title/Abstract).

After obtaining 308 records from the medical databases searched, the same terms were entered in the Google search engine and an additional seven publications were obtained. In total, there were 315 records in the database of articles. After initial review, 252 articles were excluded as they were review articles, editorials, commentaries, or letters to editors. Moreover, publications in which only abstracts were available and publications in a language other than English were also excluded. In this way, 63 articles were obtained, of which 43 were excluded because they were studies in which there was no basic data on group size, or inclusion and exclusion criteria, or after reading it turned out they contain information not related to the topic of the review. The flow diagram of the analysis is presented in Figure 3.

To assess the risk of bias and study quality in quantitative studies, the Effective Public Health Practice Project’s (EPHPP) Quality Assessment Tool for Quantitative Studies (QATQS) was used [38,39]. This tool enables quality evaluation of a wide range of study designs, including RCTs, observational studies with and without control groups, and case studies. The instrument contains eight different sections, each with multiple questions, namely: selection bias, study design, confounders, blinding, data collection methods, withdrawals and drop-outs, intervention integrity, and analyses. Each section receives a score of 1 (strong), 2 (moderate), or 3 (weak), and a final score is determined by the number of weak ratings. A strong rating is given to a study if there is no weak component score. A moderate rating is given with one weak component score. A weak rating is given with two or more component rating scores.

Additionally, a case of a 43-year-old patient suffering from BD diagnosed according to ICD-10 since the age of 34, currently presenting symptoms of moderate depression (18 points on Hamilton’s 17 points Depression Rating Scale (HDRS-17)), and treated with memantine was described.

## 5. Conclusions

The described case and the analysis of the literature, in our opinion, indicate that despite the ineffectiveness of memantine in recurrent depression, it may be an effective and safe method of augmentation in the treatment of bipolar depression. This warrants further clinical trials adding memantine to drugs that stabilize mood in bipolar depression.

## 6. Patents

The retrospective case report used data from the outpatient treatment of a 43-year-old man treated by one of the authors (M.K.) for a bipolar depression. The patient’s data were anonymized for this report, making it impossible to identify. The patient was informed about the mechanism of action of amantadine, its potential side effects, and the off-label use of drug before starting memantine. Before the treatment, the patient consented to add-on treatment with memantine.

## Figures and Tables

**Table 1:** Summary of experimental studies evaluating the antidepressant effect of memantine.

Author	Year	Behavioral Model	Animal Used	Dose of Memantine	Duration	Conclusions
Moryl et al. [18]	1993	Forced swim test	rat	5, 10, and 20 mg/kg	24, 5, and 1 h before the forced swim test	Memantine dose dependently decreased the duration of immobility time in rats. Moreover, memantine showed an antidepressant-like activity.
Rogóż et al. [19]	2002	Forced swimming test	rat	5 mg/kg and 2.5 mg/kg	24, 5, and 1 h before the forced swim test	Memantine, in combination with imipramine, fluoxetine, and venlafaxine, produced significant more enhanced antidepressant effect in rats than each of these drugs alone.
Almeida et al. [20]	2006	Forced swimming test	mice	3–10 mg/kg	30 min before the forced swim test	Acute antidepressant-like effect of memantine seemed to be dependent on the cellular signaling modulated by PKA, CaMKII, and MAPK/ERK, but not by PKC.
Réus et al. [21]	2010	Forced swimming test	rat	5, 10, and 20 mg/kg	Both 2 weeks (chronic treatment) and one single time (acute treatment)	Acute and chronic administration of memantine at all doses decreased the immobility time of rats, but only acute treatment with memantine enhanced the protein levels of BDNF in the rat hippocampus.
Quan et al. [22]	2011	21 days of exposure to chronic unpredictable stress	rat	20 mg/kg	3 weeks	Memantine improved the sucrose consumption, reversal learning, and prefrontal cortical synaptic plasticity, but impaired spatial memory, which is probably due to different extent of up-regulating NR2B receptor expression in prefrontal cortex and hippocampus in stressed rats.
Réus et al. [23]	2011	40 days of exposure to chronic mild stress	rat	20 mg/kg	1 week	Memantine normalized anhedonia, corticosterone levels, and hypertrophic adrenal gland, and increased BDNF protein levels in the rat prefrontal cortex.
Amidfar et al. [24]	2017	Forced swimming test	rat	2.5 and 5 mg/kg	2 weeks	Co-administration of antidepressant memantine with sertraline induced a more pronounced antidepressant activity than treatment with each antidepressant alone. Antidepressant properties using the combination of memantine and sertraline could be attributed to increased levels of BDNF.
Amidfar et al. [25]	2018	10 days of exposure to repeated unpredictable stress	rat	20 mg/kg	2 weeks	The administration of memantine reversed depression-like behavior and memory impairment, and significantly increased BDNF and TrkB mRNA levels in both the prefrontal cortex and hippocampus of stress exposed rats.
Li et al. [26]	2020	3 weeks corticosterone or/and copper gluconate administration	rat	20 mg/kg	2 weeks	The results of behavioral tests and cognitive function after memantine treatment were significantly normalized, and the copper concentration was decreased in all of the groups.

**Table 2:** Summary of randomized and non-randomized clinical trials (RCT and non-RCT, respectively) evaluating the efficacy of memantine in the treatment adjunctive treatment of depression in recurrent depression and bipolar depression. The risk of bias and study quality assessed with the Effective Public Health Practice Project’s Quality Assessment Tool for Quantitative Studies (QATQS), and was presented as the global rating for each publication (1—strong; 2—moderate; 3—weak).

Authors	Year	Type of Study	Sample Size	Characteristic of Participants	Intervention	Results	Conclusions	QATQS Global Rating
Zarate et al. [27].	2006	RCT	32	Subjects with major depression	Memantine (5–20 mg/day) (*n* = 16) or placebo (*n* = 16) for 8 weeks. Primary efficacy was assessed by performance on the Montgomery–Asberg Depression Rating Scale (MADRS).	The linear mixed models for total MADRS scores showed no treatment effect.	In an 8-week trial, memantine in doses of 5–20 mg/day was not effective in the treatment of major depressive disorder.	1
Ferguson and Shingleton [28]	2007	Non RCT	8	Subjects with major depression	In this 12-week subjects were treated for 4 weeks to 20 mg/d memantine. Nonresponsive patients were titrated to 30 mg/d (at week 8) or 40 mg/d (at week 10). Outcome measures were MADRS, HDRS, Clinical Global Impression-Severity of Illness and the Clinical Global Impression-Improvement Scales, Patient Global Evaluation, and Diagnostic and Statistical Manual of Mental Disorders, Fourth Edition, Major Depressive Episode Checklist.	Baseline MADRS score was 31.9 (4.45), indicative of severe depression. Seven subjects completed the study. All patients attained the target dose of 20 mg/d; 3 patients were titrated to 30 mg/d after week 8, and 2 patients were titrated to 40 mg/d after week 10. The mean dosage across all weeks was 18.1 mg. Patients improved on all efficacy measures within 1 week of treatment initiation. The mean improvement peaked at week 8 and was maintained through week 12 (MADRS, 18.5 (10.3)).	Memantine demonstrated early-onset efficacy in patients with major depression.	2
Muhonen et al. [29]	2008	RCT	58	Alcohol-dependentoutpatients with major depressivedisorder	In this 26-week study patients were treated with memantine (*n* = 29; 20 mg/day) or escitalopram (*n* = 29; 20 mg/day). Outcome measures were MADRS and Beck Depression Inventory-II for depression, Hamilton Rating Scale for Anxiety (HAM-A) and Beck Anxiety Inventory for anxiety, Consortium to Establish a Registry for Alzheimer’s Disease test battery for cognitive functions, and Social and Occupational Functioning Assessment Scale for social and occupational functions and quality-of-life measures.	Both treatments significantly reduced the baseline level of depression and anxiety according to MADRS and HAM-A, which were the primary measures (*p* < 0.0001). There was no significant difference between the memantine and escitalopram groups. Quality-of-life outcomes equally improved in both treatment groups.	These data provide new evidence for the safety and potential efficacy of memantine for major depressive disorder in patients with comorbid alcohol dependence.	1
Anand et al. [13]	2012	RCT	29	Subjects with depressive phase of bipolar disorder	Subjects on a stable dose of lamotrigine (100 mg or more) were randomized to either memantine (starting dose of 5 mg increased up to 20 mg over four weeks, then 20 mg stable dose from four to eight weeks) or matching pill placebo for eight weeks.Patients were rated on the 17-item HDRS and other behavioral measures weekly.	The 8-week repeated-measures mixed-effect model for HDRS was not significant for memantine *vs*. the placebo. Exploratory mixed-effect analyses for the first 4 weeks while the memantine dose was being titrated up every week revealed a significant decrease in HDRS scores from baseline (*p* = 0.007).	No statistically significant benefit of memantine augmentation of lamotrigine for patients with depressive phase of bipolar disorder over eight weeks was demonstrated. However, memantine had an antidepressant effect early on in the treatment while its dose was being titrated up.	1
Smith et al. [30]	2013	RCT	31	Subjects with majordepressivedisorder	Subjects were randomized to add memantine to current antidepressant treatment (flexible dose 5–20 mg/d, with all memantine group participants reaching the dose of 20 mg/d) (*n* = 15) or placebo (*n* = 16) to their existing treatment for 8 weeks. Primary outcome was measured by MADRS. Secondary outcomes included other depression and anxiety rating scales, suicidal and delusional ideation, and other adverse effects.	Participants receiving memantine did not show a statistically or clinically significant change in MADRS scores compared with the placebo over the entire study. Similarly, no substantial effect sizes favoring memantine, nor statistically significant between-group differences, were observed for the secondary efficacy outcomes.	This trial did not detect significant statistical or effect size differences between memantine and placebo augmentation among non-responders or poor responders to conventional antidepressants.	1
Strzelecki et al. [15]	2013	case report	1	49-year-old male with manic moderate depressive episode	After an ineffective use of lithium, olanzapine and antidepressant treatment with mianserin, memantine was added up to 20 mg per day for 10 weeks. The mental state was assessed using the HDRS, the Young Mania Rating Scale (YMRS), the HAM-A, the Clinical Global Inventory, the World Health Organization Quality of Life Scale and psychological tests.	After 10 weeks, the patient achieved a partial symptomatic improvement in mood, anxiety, and quality of sleep, but his activity remained insufficient. We also observed an improvement in the parameters of cognitive functioning and quality of life.	Using memantine in bipolar depression may improve mood, cognitive functioning, and quality of life.	3
Stevens et al. [16]	2013	RCT	29	Bipolar depression patients	Patients on a stable dose of lamotrigine were randomized 1:1 to receive memantine 20 mg/day and placebo. The study lasted 4–8 weeks in the memantine group and 8 weeks in the placebo group. The severity of depression was measured with the HDRS-17 scale.	There were no differences in change in the HDRS-17 score between the groups. In the group treated with memantine, an acceleration of speed of response in HDRS-17 was shown. 12 patients improved in memantine group and 6 in placebo group. No relapses were observed in the memantine group.	Memantine added to lamotrigine caused an increased speed of response compared with placebo in bipolar depression patients.	1
Omranifard et al. [31]	2014	RCT	57	Subjects with depression	Memantine (20 mg daily) or identical placebo plus citalopram for 8 weeks. Severity of depression and quality of life was evaluated using the Geriatric Depression Scale (GDS-15) HDRS and World Health Organization Quality of Life WHOQOL-BREF, respectively.	The scores of the GDS-15, HRSD, and WHO-QOL-BREF scales at baseline, 4 weeks, and 8 weeks after initiating the trial did not change significantly after the use of memantine (*p* > 0.05). There was no significant difference in mean ± SD of GDS-15, HRSD, and WHO-QOL-BREF scales among the intervention and placebo groups (*p* > 0.05).	The outcome of this clinical trial did not support the antidepressant effect of add-on memantine in elderly patients with depression receiving citalopram.	1
Lee et al. [14]	2014	RCT	232	Subjects with bipolardisorder II depression	During the 12-week study, patients undergoing regular valproic acid treatments were randomly assigned to a group: valproic acid + memantine (5 mg/day) (*n* = 115) or valproic acid + placebo (*n* = 117). The HDRS and YMRS were used to evaluate the clinical responses during weeks 0, 1, 2, 4, 8, and 12. The genotypes of the brain-derived neurotrophic factor (BDNF) Val66Met polymorphisms were determined using polymerase chain reactions plus restriction fragment length polymorphism analysis.	Both groups showed significantly decreased YMRS and HDRS scores after 12 weeks of treatment; the differences between groups were non-significant. When stratified by the *BDNF* Val66Met genotypes, significantly greater decreases in HDRS scores were found in the VPA + memantine group in patients with the Val Met genotype (*p* = 0.004).	The effectiveness of memantine in the treatment of depression depended on polymorphisms in the *BDNF* gene.	1
Amidfar et al. [32]	2017	RCT	66	Subjects with moderate-to-severe major depressivedisorder	6 weeks of treatment with either memantine (20 mg/day) plus sertraline (200 mg/day) or placebo plus sertraline (200 mg/day). Patients were evaluated using HDRS at baseline and at weeks 2, 4, and 6.	A significantly greater improvement was seen at all three follow-up sessions, as well as significantly greater response rates at weeks 4 and 6, in the memantine group. Significantly more early improvements and more rapid response to treatment were observed in the memantine group. A significant reduction was observed in the HDRS score from baseline to the study endpoint in both the memantine and placebo groups.	A 6-week course of treatment with memantine as an adjunct to sertraline showed a favorable safety and efficacy profile in patients with major depressive disorder.	1
Lavretsky et al. [33]	2020	RCT	62	Older adults with depression and subjective memory complaints	Escitalopram + memantine (ESC/MEM) compared with escitalopram + placebo (ESC/PBO) for 6 months. The primary outcome was a change in depression, as assessed by HDRS post-treatment (at 6 months). Remission was defined as HDRS ≤6; naturalistic follow-up continued until 12 months.	The mean daily escitalopram dose was 11.1 mg (SD = 3.7; range: 5–20 mg). The mean daily memantine dose was 19.3 mg (SD = 2.6; range 10–20 mg). The remission rate within ESC/MEM was 45.8% and 47.9%, compared with 38.3% and 31.9% in ESC/PBO, at 3 and 6 months, respectively. Both groups improved significantly on the HAM-D at 3, 6, and 12 months, with no observed between-group differences. ESC/MEM demonstrated greater improvement in delayed recall and executive functioning at 12 months compared with ESC/PBO.	The combination of memantine with escitalopram was well tolerated and was as effective as escitalopram and placebo in improving depression using HAM-D. The combination of memantine and escitalopram was significantly more effective than escitalopram and the placebo at improving cognitive outcomes at 12 months.	1

**Table 3:** Drugs used in the treatment of a patient with memantine treated with bipolar depression, with severity of depression measured with HDRS-17.

Visit Time	HDRS-17 (Points)	Treatment (Daily Dose)
11/2018	18	quetiapine 300 mg
01/2019	18	quetiapine 300 mg + lamotrigine 150 mg
03/2019	19	lamotrigine 150 mg + aripiprazol 7.5 mg
05/2019	18	lamotrigine 150 mg + aripiprazol 7.5 mg
07/2019	19	lamotrigine 150 mg + valproic acid 1500 mg
10/2019	18	lamotrigine 150 mg + valproic acid 1500 mg + amantadine 100 mg/200 mg
10/2019	15	lamotrigine 150 mg + valproic acid 1500 mg + amantadine 200 mg
11/2019	17	lamotrigine 150 mg + valproic acid 1500 mg + memantine titrated-up every 3 days (5 mg/10 mg/15 mg/20 mg)
11/2019	16	lamotrigine 150 mg + valproic acid 1500 mg + memantine 20 mg
12/2019	13	lamotrigine 150 mg + valproic acid 1500 mg + memantine 20 mg
12/2019	11	lamotrigine 150 mg + valproic acid 1500 mg + memantine 20 mg
01/2020	9	lamotrigine 150 mg + valproic acid 1500 mg + memantine 20 mg
02/2020	13	lamotrigine 150 mg + valproic acid 1500 mg + memantine 5 mg
03/2020	7	lamotrigine 150 mg + valproic acid 1500 mg + memantine 10 mg

## Data Availability

Data supporting reported results on the treatment of the patient can be found in the author’s (M.K.) medical record, and can be obtained upon request from corresponding author.
